# Prostate MRI using a rigid two-channel phased-array endorectal coil: comparison with phased array coil acquisition at 3 T

**DOI:** 10.1186/s40644-022-00453-7

**Published:** 2022-03-16

**Authors:** Sara Lewis, Aasrith Ganti, Pamela Argiriadi, Ally Rosen, Stefanie Hectors, Sahar Semaan, Christopher Song, Steve Peti, Maxwell Segall, Kezia George, Vaneela Jaikaran, Sebastian Villa, David Kestenbaum, Nicholas Voutsinas, John Doucette, Ashutosh Tewari, Ardeshir R. Rastinehad, Bachir Taouli

**Affiliations:** 1 Department of Diagnostic, Molecular and Interventional Radiology, Icahn School of Medicine at Mount Sinai, Box 1234, New York, NY 10029 USA; 2 BioMedical Engineering and Imaging Institute, Icahn School of Medicine at Mount Sinai, New York, NY USA; 3 In Vivo Corporation, Philips Healthcare, Gainesville, FL USA; 4 Environmental Medicine & Public Health, Icahn School of Medicine at Mount Sinai, New York, NY USA; 5 Department of Urology, Icahn School of Medicine at Mount Sinai, New York, NY USA; 6 Department of Urology, Lenox Hill Hospital, New York, NY USA

**Keywords:** Prostate cancer, Magnetic resonance imaging, Endorectal coil, Signal to noise ratio (SNR), Image quality

## Abstract

**Background:**

To compare image quality, lesion detection and patient comfort of 3T prostate MRI using a combined rigid two-channel phased-array endorectal coil and an external phased-array coil (ERC-PAC) compared to external PAC acquisition in the same patients.

**Methods:**

Thirty three men (mean age 65.3y) with suspected (*n* = 15) or biopsy-proven prostate cancer (PCa, *n* = 18) were prospectively enrolled in this exploratory study. 3T prostate MRI including T2-weighted imaging (T2WI) and diffusion-weighted imaging (DWI) was performed using an ERC-PAC versus PAC alone, in random order. Image quality, lesion detection and characterization (biparametric PI-RADSv2.1) were evaluated by 2 independent observers. Estimated signal-to-noise ratio (eSNR) was measured in identified lesions and the peripheral zone (PZ). Patient comfort was assessed using a questionnaire. Data were compared between sequences and acquisitions. Inter/intra-observer agreement for PI-RADS scores was evaluated.

**Results:**

Twenty four prostate lesions (22 PCa) were identified in 20/33 men. Superior image quality was found for ERC-PAC compared to PAC for T2WI for one observer (Obs.1, *p* < 0.03) and high b-value DWI for both observers (*p* < 0.05). The sensitivity of PI-RADS for lesion detection for ERC-PAC and PAC acquisitions was 79.2 and 75% for Obs.1, and 79.1 and 66.7%, for Obs.2, without significant difference for each observer (McNemar *p*-values ≥0.08). Inter−/intra-observer agreement for PI-RADS scores was moderate-to-substantial (kappa = 0.52–0.84). Higher eSNR was observed for lesions and PZ for T2WI and PZ for DWI using ERC-PAC (*p* < 0.013). Most patients (21/33) reported discomfort at ERC insertion.

**Conclusion:**

Despite improved image quality and eSNR using the rigid ERC-PAC combination, no significant improvement in lesion detection was observed, therefore not supporting the routine use of ERC for prostate MRI.

**Supplementary Information:**

The online version contains supplementary material available at 10.1186/s40644-022-00453-7.

## Introduction

Multiparametric MRI (mpMRI) of the prostate, using a combination of T2-weighted imaging (T2WI), diffusion weighted imaging (DWI) and dynamic contrast enhanced (DCE)-MRI, is highly valuable for the assessment of prostate cancer (PCa) demonstrating value for risk stratification and informing management decisions [1, 2]. Prostate mpMRI using a 3 T system and an external phased array body coil (PAC) is the current practice standard [3–5] given the recent software and hardware improvements resulting in gains in signal-to-noise ratio (SNR) attributed to scanning at higher field strength, the use of PACs with a greater number of receiver elements and improved pulse sequence techniques. Image quality is a major factor impacting the performance of mpMRI to accurately detect, characterize and stage PCa [5], and there are ongoing efforts to standardize imaging protocols [4].

Endorectal coils (ERC) were historically used at 1.5 T given gains in signal-to-noise ratio (SNR), however, are still not widely used, likely due to the anticipated discomfort associated with the ERC, increased costs and the lack of expertise at all centres. Mixed results comparing image quality and lesion detection have been reported when comparing PAC to balloon-inflatable ERC at 3 T [6–12]. Recent hardware and software developments have fueled interest in newer multichannel endoluminal devices to achieve such gains in SNR [13, 14]. A new rigid two-channel receive ERC has shown improved SNR and image quality on T2WI compared to the balloon-inflatable ERC at 1.5 T, with less prostate deformation [15, 16]. These rigid coils have a smaller diameter compared to an inflated coil. Disposable rigid ERC devices have also been developed with equivalent coil profiles compared to the reusable devices. The use of the rigid ERC combined with external PAC and parallel imaging provides further opportunities to improve SNR and possibly reduce scan time, potentially translating to improved image quality and lesion detection.

The purpose of this study is to compare image quality, lesion detection and patient comfort of 3 T prostate MRI using a combined rigid two-channel phased-array ERC and an external PAC (ERC-PAC) compared to external PAC acquisition in the same patients.

## Methods

### Patients

This prospective, single-centre study was approved by our local institutional review board and signed informed consent was obtained in all subjects. A formal power analysis for sample size calculation was not performed given the exploratory nature of the study. Thirty-three consecutive men (mean age 65.3 ± 7.7y; range 48–78y) who underwent mpMRI of the prostate at 3 T were enrolled from 2/2017–8/2018. Inclusion criteria included men with biopsy-proven PCa on MRI-ultrasound (US) fusion guided prostate biopsy (UroNav, Philips Healthcare, Best NL), elevated serum PSA or abnormal digital rectal exam. Methodology for MRI-US fusion guided prostate biopsy has been previously reported [17]. Exclusion criteria included men with previously treated PCa. No patients were excluded based on these criteria. Clinical, laboratory and pathology data are shown in Table 1.

**Table 1:** Characteristics of the study population (*n*=33). Continuous variables such as age, serum PSA, and PSAD are expressed as means ± standard deviation, with range of values provided

Parameter	
Age (y)	65.3 ± 7.7 (48-78)
Race/Ethnicity	
Caucasian	22
African American	7
Hispanic	2
Asian	2
Serum PSA (ng/mL)	9.9 ± 6.5 (0.82-36.7)
Serum PSAD^a^ (ng/mL/g)	0.15 ± 0.09 (0.04-0.41)
Pathology Results^b^	22 total PCa lesions in 18 patients
Negative biopsy	14
Gleason 3+3	11
Gleason 3+4	6
Gleason 4+3	4
Gleason 7^c^	1

^a^*PSAD* Prostate specific antigen density, defined as PSA/prostate volume calculated with MRI

^b^One patient did not undergo prostate biopsy and therefore this information was not available

^c^One patient underwent prostate biopsy at an outside institution and the Gleason score was reported as “Gleason 7”

### MRI technique

**Fig. 1:**
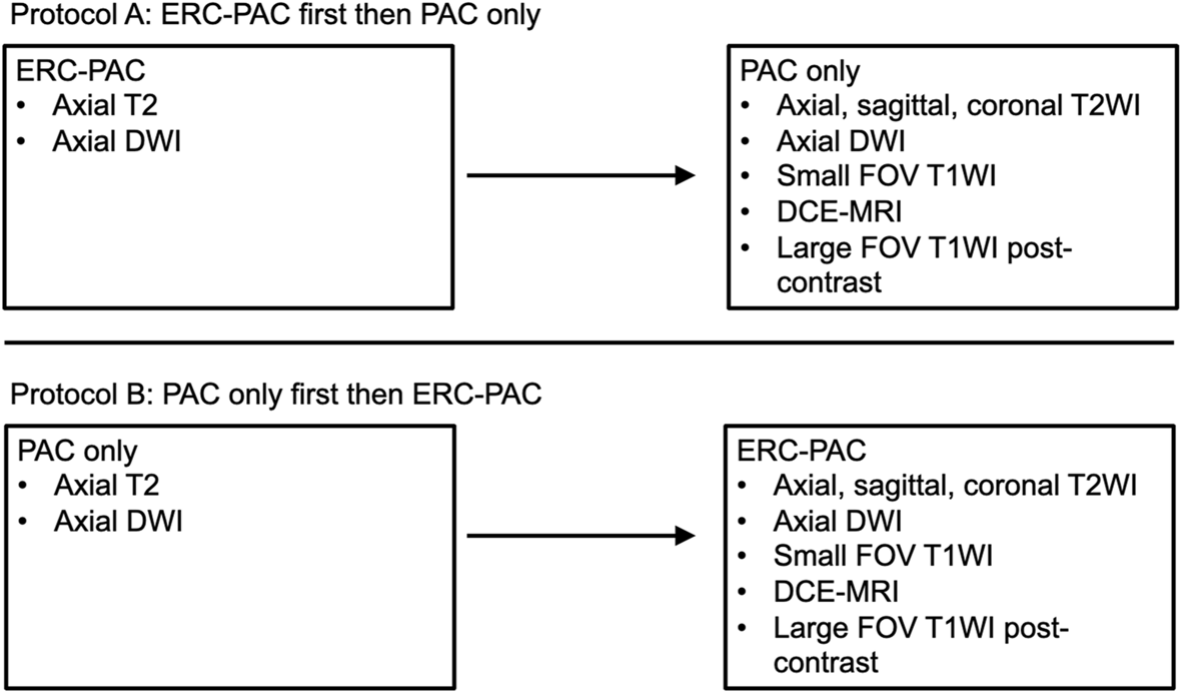
Patients were randomized to one of two protocols, which determined the order of image acquisition. A total of *n* = 18 (55%) and *n* = 15 (45%) of patients were randomized to protocol A and B, respectively

All patients underwent prostate 3 T mpMRI (Skyra, Siemens) using a rigid two-channel receive phased-array ERC (Sentinelle, InVivo Corporation, Philips Healthcare) combined with a PAC (18-elements) (ERC-PAC) vs. PAC only. All patients were placed in the head-first supine position on the MRI table. Patients were randomized to one of two protocols: Protocol A (axial T2WI and DWI using combined ERC-PAC; followed by axial/coronal/sagittal (multiplanar) T2WI, axial DWI and axial DCE-MRI using PAC; *n* = 18) or Protocol B (axial T2WI and DWI with PAC; followed by multiplanar T2WI, axial DWI and axial DCE-MRI using ERC-PAC; *n* = 15) (Fig. 1). The sequence parameters were consistent with published guidelines [18] and were identical for both the PAC and ERC-PAC acquisitions (Table 2). Apparent diffusion coefficient (ADC) maps were calculated using a monoexponential fitting based on the three obtained b-values (b = 50, 1000, 2000 s/mm^2^). Because the use of an ERC can result in high signal intensity artefacts close to the coil, a pre-scan normalizer filter was used to homogenize signal intensities over the whole scan volume [9]. The gadolinium based contrast agent used for all cases was gadoterate meglumine (Dotarem, Guerbet, France). All patients performed a bowel preparation consisting of a Fleet’s™ enema prior to MRI. Bowel motility was suppressed with 1 mg of intravenous glucagon administered intravenously prior to mpMRI (except in diabetic patients).

**Table 2:** 3T bpMRI protocol with the sequence parameters for T2WI and DWI are shown below. Only the axial T2WI and DWI were repeated using the ERC-PAC and PAC acquisitions. DCE-MRI was performed only once during the exam, using either ERC-PAC or PAC, depending on the order of randomization

Sequence	TR	TE	FA	SA	GRAPPA	AT	ST (mm)	FOV	Matrix
Axial T2WI	6460	116	137	2	2	5:25	3	180x180	320x320
Axial DWI*	8700	72	90	1,4,8	2	6:24	3	250x250	114x114

*Abbreviations*: *TR* Time to repetition, *TE* Time to echo, *FA* Flip angle, *SA* Signal averages, *GRAPPA* Generalized Autocalibrating Partial Parallel Acquisition factor, *AT* Acquisition time (min:sec), *ST* Slice thickness, and *FOV* Field of view.

*b-values used were 50,1000, 2000

### Coil design and insertion

Rigid two-channel receive ERC devices used included a reusable coil (*n* = 20) and a disposable coil (*n* = 13). Details of the rigid ERC coil design are summarized in Supplemental Material and an image of the reusable device is shown in Supplemental Fig. 1. The reusable and disposable coils are treated without distinction in this manuscript for purposes of data analysis. High-level disinfection was achieved through liquid chemical submersion for both the reusable and disposable coils. A sterile condom cover was placed on the sterilized rigid ERC at the time of scanning. A thin layer of water-soluble gel was applied to the condom cover sparingly at insertion. The ERC was inserted into the rectum with the patient in the supine position. The ERC was stabilized using a locking articulated mechanical arm attached to a tabletop support.

### Qualitative image analysis

Two independent fellowship trained abdominal radiologists (Observer 1, with 11 years of experience and Observer 2, with 13 years of experience, in interpreting prostate MRI) reviewed the axial T2WI, DWI and corresponding ADC maps in two separate reading sessions for the ERC-PAC and PAC acquisitions. The observers were blinded to all clinical, laboratory and pathology information. To avoid recall bias, the time interval between the reading sessions was 6 weeks and the image sets were presented in random order. Since DCE-MRI was performed once per MRI exam (either ERC-PAC or PAC, depending on the randomization) and intra-individual comparison could not be performed, the DCE-MRI data was therefore not assessed. Each of the following features were assessed on a 5-point Likert scale on T2WI, DWI and ADC images: A) image sharpness/structure detail, B) motion artefact, C) DWI anatomic distortion, D) ghosting/susceptibility artefacts, E) ERC-related phase artefacts and F) overall image quality [19] **(**Table 3**)**.

**Table 3:** Image quality (IQ) assessed by two independent observers on axial T2WI and DWI/ADC for both combined rigid phased-array endorectal coil and an external phased-array coil (ERC-PAC) and PAC acquisitions

**Image sharpness/structure detail**	
◦ Definition: Clear depiction of prostate edge or degree of blurring, clarity of border delineation, peripheral-transition zone boundary, and the extent of lesion definition and internal morphologic features	
◦ Scores: 1, non-diagnostic; 2, poor; 3, satisfactory; 4, good; and 5, excellent sharpness/detail.	
**Motion artifact**	
◦ Definition: Presence and severity of image blurring due to patient motion	
◦ Scores: 1, non-diagnostic; 2, severe; 3, moderate; 4, mild; and 5, motion free	
**Anatomic distortion**	
◦ Definition: Distortion in size, profile, or orientation of the prostate using T2W as a reference	
◦ Scores: 1, non-diagnostic; 2, severe; 3, moderate; 4, mild; and 5, no distortion	
**Ghosting/susceptibility artifacts**	
◦ Definition: Displaced reduplications of image or structure that propagate in phase encoding direction	
◦ Scores: 1, non-diagnostic; 2, severe; 3, moderate; 4, mild; and 5, no ghosting/susceptibility	
**Coil related phase artifacts**	
◦ Definition: Displaced reduplications of the endorectal coil that propagate in phase encoding direction	
◦ Scores: 1, non-diagnostic; 2, severe; 3, moderate; 4, mild; and 5, no coil artifact	
**Overall image quality**	
◦ Scores: 1, non-diagnostic; 2, poor; 3, satisfactory; 4, good; and 5, excellent image quality	

### Lesion detection and characterization

During the same reading sessions, the observers evaluated the axial T2WI and DWI/ ADC images acquired with the ERC-PAC and PAC for lesion detection and characterization. A maximum of 3 lesions per patient were recorded. Lesion size, as measured on axial T2WI, and lesion location were recorded on a diagram of prostate anatomy. Prostate lesions were evaluated according to the PI-RADS version 2.1 scoring system [20]. Since DCE-MRI was only performed once during the exam, these images were not evaluated in order to prevent any bias due to this additional information, and therefore the “bi-parametric” PI-RADS scoring algorithm using T2WI and DWI were used [20]. Lesion conspicuity, as defined as the ability to distinguish prostate lesions from normal gland tissue, was assessed using the following scale: 1, not visualized; 2, poor conspicuity; 3, moderate conspicuity, 4, good conspicuity; and 5, excellent conspicuity.

### Quantitative image analysis

One observer (Observer 3, a trained radiologist with 9 years of experience in interpreting prostate MRI) placed an elliptic region-of-interest (ROI) within prostate lesions identified at the consensus reference standard (see below) and normal appearing tissue in the peripheral zone (PZ) on the T2WI and b2000 DWI images of each patient avoiding artefacts. The minimum ROI size was 10 mm^2^. Quantitative analysis with measurement of signal intensity (SI) was performed using the Picture Archiving and Communication System (PACS, GE Centricity v6, Chicago, IL). Estimated SNR [eSNR as mean (SI)/standard deviation (SD) of the SI] in the ROI on T2WI and b2000 DWI images was calculated [19, 21]. Lesion-to-PZ contrast ratio (CR) was calculated for T2WI and b2000 DWI for both acquisitions as: [(SI_lesion-SI_PZ)/(SI_lesion+SI_PZ)].

### Reference standard

A consensus panel, consisting of Observers 3 and 4 (the latter a urologist with 13 years of experience), established the panel reference standard for prostate lesions in all patients. The consensus panel had access to all available clinical, laboratory, histopathologic and imaging data, including both ERC-PAC and PAC acquisitions and DCE-MRI data. Prostate MRI interpretation was also based on PI-RADS version 2.1 scoring system, using T2W, DWI and DCE sequences [20]. Lesions were considered to be a match between MRI and pathology when occurring within the same location and laterality of the prostate gland, and only the matched lesions were included for the final lesion analysis.

### Patient questionnaire

Patient comfort was assessed at the time of ERC insertion and during the exam for the ERC-PAC and PAC acquisitions (1, very uncomfortable; 2, somewhat uncomfortable; 3, neutral; 4, somewhat comfortable; and 5, very comfortable) on a questionnaire performed at the end of the exam. The patients were also asked whether they would prefer an MRI exam with the ERC if superior diagnostic accuracy was found (1, definitely yes; 2, probably yes; 3, probably no; and 4, definitely no).

### Statistical analysis

Clinical and demographic data were summarized using descriptive statistics, expressed as mean ± standard deviation (SD). Qualitative and quantitative data for sequences were compared between acquisitions using the Wilcoxon matched pairs signed-rank test. Differences in eSNR data acquired using the different ERC coil designs were also assessed. The McNemar test was used to assess differences in sensitivity and specificity for each observer for the ERC-PAC and PAC acquisitions for each observer. Sensitivity, specificity, positive predictive values (PPV), and negative predictive values for lesion detection were calculated, using the reference standard assessment. Inter- and intra-reader agreement for PI-RADS classification was assessed using kappa statistics. Agreement was calculated only for lesions that were detected by both observers and both coil designs. *P* ≤ 0.05 was considered statistically significant. All statistical tests were conducted using Graph-Pad Prism v8.2.0 (GraphPad Software, La Jolla California USA) and SPSS (IBM, Armonk NY).

## Results

### Patients

Thirty-three patients who matched the eligibility criteria were included. Mean patient age was 65.3 ± 7.7y (range 48–78y). Mean PSA level at the time of mpMRI was 9.9 ± 6.5 ng/ml (range, 0.8–36.7 ng/mL) and mean PSA density (PSAD) was 0.15 ± 0.09 (ng/mL)/mL.

### Pathology results

Thirty-two patients had transrectal ultrasonography (TRUS)-guided prostate biopsy before or after prostate MRI (median interval from biopsy to the time of MRI 126 ± 511 days). One patient did not have biopsy correlation and underwent MRI for elevated PSA. Fourteen patients had negative prostate biopsies. A total of 22 PCa were found in 18 patients at biopsy; four patients had 2 lesions and the remaining 14 patients had a single lesion at biopsy. The Gleason score distribution for the PCa lesions (*n* = 22) was as follows: 3 + 3 (*n* = 11), 3 + 4 (*n* = 6), 7 (*n* = 1; breakdown of Gleason score was not provided in the pathology report), and 4 + 3 (*n* = 4). The characteristics of the study population are summarized in Table 1. There was no significant difference in median PSA or PSAD for patients without and with PCa (*p* = 0.84 and *p* = 0.15, respectively). Only one patient from the study cohort underwent subsequent prostatectomy, which confirmed Gleason 3 + 4 PCa at the left apex.

### Reference standard

Twenty four lesions (5 PI-RADS 3, 14 PI-RADS 4 and 5 PI-RADS 5 lesions) were identified in 20 patients. Lesions were located in the PZ (*n* = 18), TZ (*n* = 4) and both PZ/TZ (*n* = 2). There were 2 patients with negative prostate biopsies in whom lesions were identified at mpMRI. There were no patients with negative MRI in which a lesion was identified at biopsy. Mean lesion size at MRI was 12 ± 6 mm (range 6–29 mm).

### Image quality assessment

Superior image quality was found for ERC-PAC for T2WI for observer 1 only (*p* < 0.03) and high b-value DWI for both observers (*p* < 0.05) compared to PAC. Observer 2 found lower image quality for low b-value DWI using ERC-PAC (*p* < 0.05) **(**Table 4**)**. Mixed results were found for ADC maps, as observer 1 reported higher image quality for ERC-PAC (*p* < 0.02) while observer 2 reported lower image quality for ERC-PAC (*p* < 0.02). Observer 2 rated ERC-related phase artefacts to be worse for DWI compared to T2WI (*p* = 0.0137 for b50 and *p* = 0.0459 for b2000).

**Table 4:** Image quality analysis between the combined rigid two-channel phased-array endorectal coil and an external phased-array coil (ERC-PAC) and the external PAC datasets for 2 independent observers. Mean and standard deviation for each image quality parameter are shown and significant values are highlighted in bold

	Feature	Observer 1			Observer 2		
		PAC	ERC-PAC	p	PAC	ERC-PAC	***p***
**T2WI**	Sharpness/detail	3.7 ± 0.6	4.1 ± 0.9	**0.03**	3.7 ± 1.0	3.7 ± 0.9	0.95
	Motion	3.7 ± 0.7	4.0 ± 0.8	0.07	3.4 ± 0.9	3.4 ± 0.7	0.95
	Overall Quality	3.6 ± 0.7	4.0 ± 0.8	**0.03**	3.5 ± 0.9	3.5 ± 0.8	0.89
**DWI low b-value (b50)**	Sharpness/detail	3.8 ± 0.4	4.0 ± 0.4	0.17	4.0 ± 0.5	3.8 ± 0.6	0.34
	Motion	3.8 ± 0.4	4.0 ± 0.4	0.23	4.0 ± 0.3	3.8 ± 0.7	0.27
	Distortion	3.9 ± 0.4	4.0 ± 0.4	0.30	3.9 ± 0.5	3.5 ± 0.7	**0.02**
	Ghosting/susceptibility	3.9 ± 0.3	4.0 ± 0.4	0.45	4.3 ± 0.7	3.9 ± 0.9	**0.02**
	Overall quality	3.9 ± 0.3	4.0 ± 0.4	0.13	3.9 ± 0.3	3.7 ± 0.6	**0.05**
**DWI high b-value (b2000)**	Sharpness/detail	3.8 ± 0.4	4.1 ± 0.6	**0.05**	2.8 ± 0.7	3.5 ± 0.7	**<0.001**
	Motion	3.8 ± 0.4	4.1 ± 0.5	**0.02**	2.9 ± 0.6	3.4 ± 0.7	**0.02**
	Distortion	3.8 ± 0.5	4.1 ± 0.5	**0.01**	3.2 ± 0.7	3.4 ± 0.8	0.59
	Ghosting/susceptibility	3.8 ± 0.5	4.1 ± 0.5	**0.01**	3.5 ± 0.9	3.7 ± 0.9	0.63
	Overall quality	3.8 ± 0.4	4.1 ± 0.5	**0.02**	3.0 ± 0.7	3.4 ± 0.7	**0.02**
**ADC**	Sharpness/detail	3.7 ± 0.5	4.1 ± 0.5	**0.01**	3.6 ± 0.6	3.2 ± 0.6	**0.01**
	Motion	3.8 ± 0.4	4.1 ± 0.5	**0.01**	3.6 ± 0.5	3.2 ± 0.7	**0.02**
	Distortion	3.8 ± 0.5	4.1 ± 0.4	**0.001**	3.7 ± 0.5	3.4 ± 0.6	0.07
	Ghosting/susceptibility	3.8 ± 0.5	4.1 ± 0.4	**0.01**	4.0 ± 0.6	3.5 ± 0.9	**0.002**
	Overall quality	3.8 ± 0.5	4.1 ± 0.4	**0.002**	3.6 ± 0.5	3.2 ± 0.7	**0.003**

*Abbreviations*: *DWI* Diffusion weighted imaging, *ADC* Apparent diffusion coefficient

### Lesion detection and characterization

Observer 1 identified 27 lesions using ERC-PAC with PI-RADS scores of 3 (*n* = 1), 4 (*n* = 22) and 5 (*n* = 4) and 28 lesions using PAC with PI-RADS scores of 3 (*n* = 1), 4 (*n* = 22) and 5 (*n* = 5). For Observer 1, the sensitivity, specificity, positive predictive value, and negative predictive value, of bi-parametric PI-RADS v2.1 score for lesion detection were 79.2%, 65.2%, 70.4%, 75.0% and 75%, 56.5%, 64.3%, 68.4% for the ERC-PAC and PAC acquisitions, respectively. There was no difference in sensitivity (McNemar *p* = 0.56) or specificity (McNemar *p* = 0.48) or Observer 1 between ERC-PAC and PAC acquisitions.

**Fig. 2:**
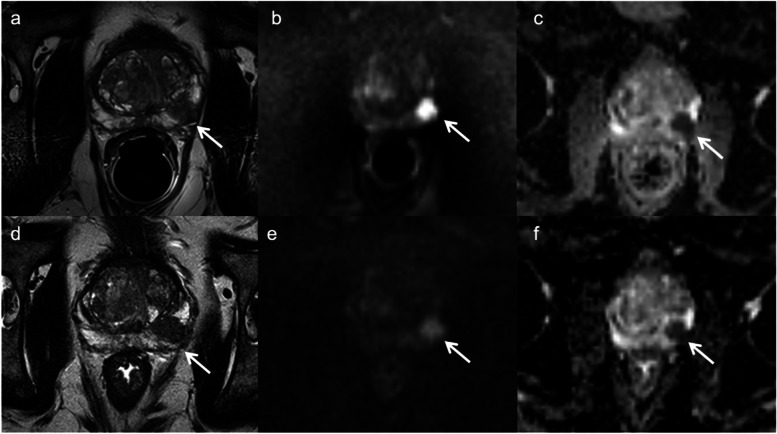
66-year old man (PSA of 9.03 ng/mL) who underwent MRI using a combined rigid two-channel phased-array endorectal coil and an external phased-array coil (ERC-PAC) (top row) and external PAC only acquisition (bottom row) with axial T2WI (**a**, **d**), DWI (b2000) (**b**, **e**) and ADC (**c**, **f**). A 15 mm PI-RADS 5 lesion (arrow) was identified in left mid-gland peripheral zone. Biopsy revealed Gleason 4 + 3 cancer in this location. Superior lesion detection and conspicuity are noted for ERC-PAC images. For this lesion, the estimated signal-to-noise (eSNR) for ERC-PAC vs. PAC alone were 11.6 vs 5.5  for T2WI and 36.3  vs 5.8 for b2000 DWI, respectively

Observer 2 identified 25 lesions using ERC-PAC with PI-RADS scores of 3 (*n* = 5), 4 (*n* = 16) and 5 (*n* = 4) and 19 lesions using PAC with PI-RADS scores of 2 (*n* = 1), 3 (*n* = 1), 4 (*n* = 11) and 5 (*n* = 6). For Observer 2, the sensitivity, specificity, positive predictive value, and negative predictive value of bi-parametric PI-RADS score for lesion detection were 79.2%, 64.7%, 76.0%, 68.8% and 66.7%, 78.6%, 84.2%, 59.7% for the ERC-PAC and PAC acquisitions, respectively. There was no difference in sensitivity (McNemar *p* = 0.08) or specificity (McNemar *p* = −-, indicating perfect agreement) or Observer 2 between ERC-PAC and PAC acquisitions. There were no significant differences in overall lesion conspicuity between ERC-PAC and PAC acquisitions for T2WI and DWI for both observers (all *p*-values > 0.17). Figure 2 illustrates an example of lesion conspicuity for a peripheral zone prostate cancer confirmed at biopsy in one patient.

### Inter- and intra-observer analysis

There was substantial agreement between observers for PI-RADS scores for T2WI, DWI and bi-parametric PI-RADS score (all k = 0.65, 95%CI 0.41–0.88) for ERC-PAC. Substantial inter-observer agreement was also found for PAC: T2WI (k = 0.60, 95% CI 0.36–0.84), DWI (k = 0.60, 95% CI 0.36–0.84) and bi-parametric PI-RADS score (k = 0.68, 95% CI 0.45–0.91. There was moderate to excellent intra-observer agreement for PI-RADS scores obtained from ERC-PAC and PAC acquisitions for Observer 1 (k = 0.52, 95% CI 0.27–0.77) and Observer 2 (k = 0.84, 95% CI 0.67–1.00).

### Quantitative analysis

**Fig. 3:**
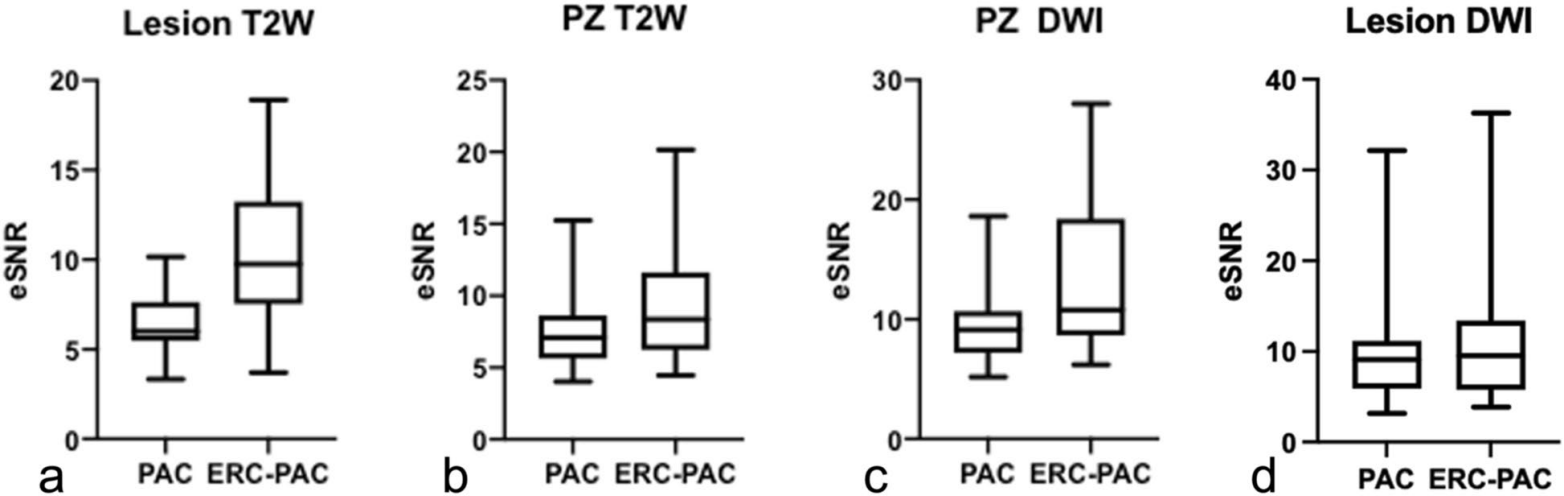
Boxplot distribution of estimated signal-to-noise ratio (eSNR) for sequences performed using a combined rigid two-channel phased-array endorectal coil and an external phased-array coil (ERC-PAC) and external PAC only acquisition. Higher eSNR was observed for lesions for T2W (**a**, *p* < 0.0001), PZ for T2W (**b**, *p* < 0.013) and PZ for DWI (**c**, *p* < 0.01) when using ERC-PAC compared to PAC (shown below in boxplots). There was no difference in eSNR for lesions on DWI (**d**, *p* = 0.26)

Significantly higher eSNR was observed for lesions and PZ for T2WI and PZ for DWI when using ERC-PAC compared to PAC (Fig. 3; Table 5). The average increase in eSNR for sequences performed using ERC-PAC compared to PAC ranged from 27.7 ± 46.4% for PZ and 57.8 ± 49.3% for lesions on T2WI (Table 5). There was no difference in lesion-to-PZ contrast ratios (CR) for T2WI (− 0.31 ± 0.14 vs. -0.33 ± 0.13, *p* = 0.60), while there was higher CR for b2000 DWI (0.22 ± 0.26 vs. 0.15 ± 0.15, *p* = 0.0006) for ERC-PAC vs. PAC.

**Table 5:** Comparison of estimated mean signal-to-noise ratio (eSNR) for T2WI and b2000 DWI for prostate lesions and peripheral zone (PZ). Mean and standard deviation for each eSNR value are shown and significant values are highlighted in bold. The mean %∆ eSNR was calculated as [eSNR (ERC-PAC)-eSNR (PAC)]/[eSNR (PAC)]x100

	eSNR PAC	eSNR ERC-PAC	***p***	Mean %∆ eSNR
T2W				
Lesions	6.5 ± 1.7	10.1 ± 3.8	**<0.0001**	57.8 ± 49.3
PZ	7.4 ± 2.2	9.0 ± 3.6	**<0.013**	27.7 ± 46.4
DWI (b2000)				
Lesions	9.8 ± 6.2	12.1 ± 8.7	0.26	57.7 ± 141.8
PZ	9.8 ± 3.5	13.7 ± 6.2	**<0.01**	55.1 ± 87.2

### Patient comfort

**Fig. 4:**
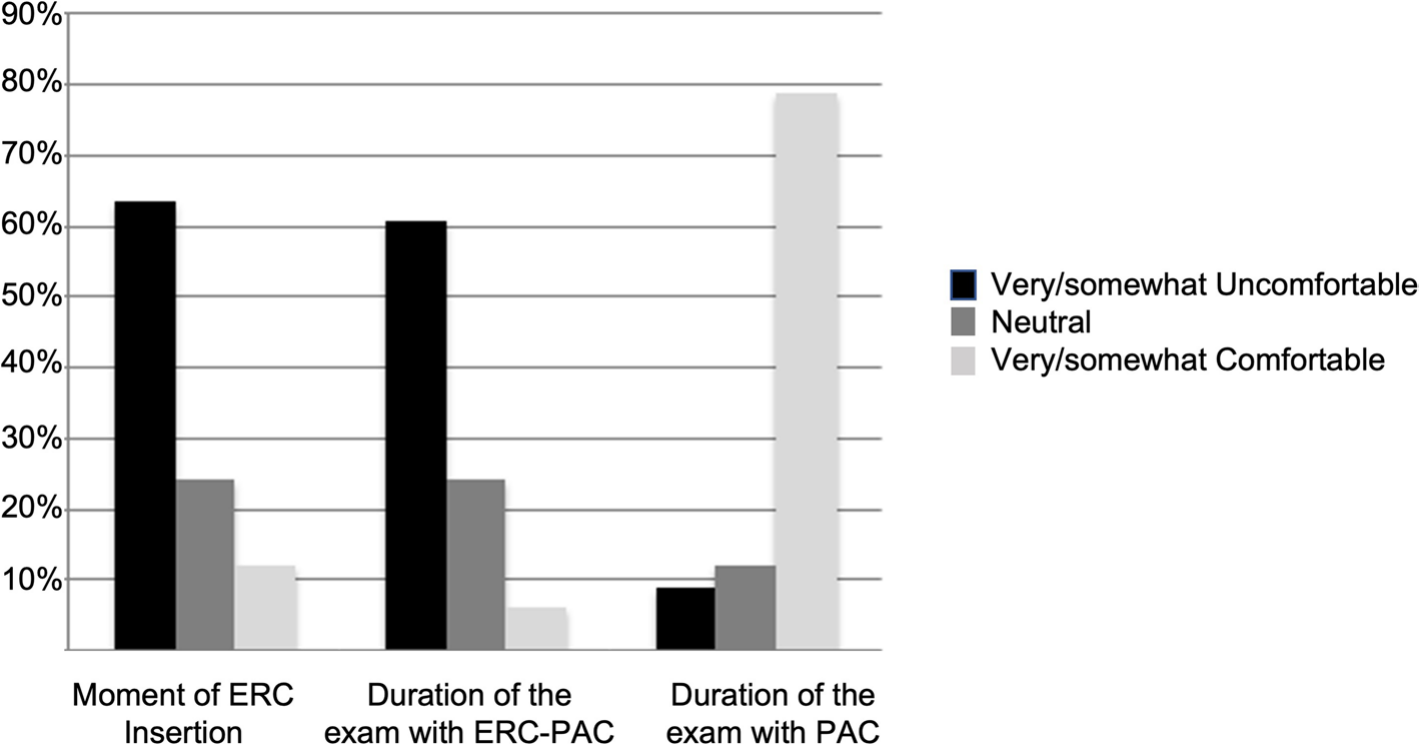
Patient comfort for MRI sequences obtained with and without the ERC was assessed using a questionnaire

The majority of patients reported feeling either “somewhat uncomfortable” or “very uncomfortable” at the time of ERC insertion (*n* = 21/33, 63.6%). The majority of patients reported feeling “somewhat uncomfortable” or “very uncomfortable” in *n* = 11/33 (33%) or “neutral” *n* = 12/33 (36.4%) during the exam with the ERC in place. The majority of patients reported feeling comfortable during the exam with the PAC only (*n* = 26/33, 78.8%). However, the vast majority of patients (97% = 32/33) reported definitely or probably choosing to have an exam with ERC-PAC if superior diagnostic accuracy is shown **(**Fig. 4**)**.

## Discussion

Currently, the most widely used approach for mpMRI of the prostate for tumour detection, characterization and staging uses a 3 T MRI platform with a PAC. While few prior studies have suggested that the addition of an ERC using a balloon inflatable coil demonstrates improved image quality and performance of prostate MRI at 3 T [6, 7, 10], there is no clear consensus regarding the potential benefits of the use of the ERC with 3 T MRI in clinical practice [4]. Furthermore, there is a paucity of data investigating the difference in diagnostic performance without and with the ERC in the same patients [8, 9]. To the best of our knowledge, there is no published data evaluating rigid ERC designs combined with PAC (ERC-PAC) compared to PAC alone, to assess diagnostic performance and patient comfort.

Although the advantages afforded by the ERC were not fully utilized as sequence parameters were deliberately kept identical between ERC-PAC and PAC acquisitions, we found that despite improvements in eSNR and image quality on T2WI and high b-value DWI for ERC-PAC, there was no difference in diagnostic performance between coil set ups, with considerable discomfort reported in most patients.

Contextualizing our results to prior studies requires careful consideration of imaging acquisition parameters, and for the purposes of this discussion, we will primarily focus on published studies that closely matched acquisition parameters. As expected, we observed higher eSNR on T2WI and DWI using ERC-PAC compared to PAC. These findings are consistent with a prior study of 25 patients imaged using a balloon inflatable ERC-PAC compared to PAC, in which the SNR on ADC was significantly higher in PZ [22]. The greatest gains in eSNR were found on ADC maps derived from higher b-value DWI (b1500) compared to b1000 (25.3% vs. 13.21% difference, respectively) [22]. There was no difference in SNR for T2W images acquired with PAC versus ERC-PAC in a prior study where the T2WI sequence parameters closely matched [11]. Interestingly, a recent study found that SNR on T2WI using a solid reusable rigid ERC (different than the coil design we used) was higher compared to that of the balloon ERC (*p* < 0.001), with SNR improvements ranging from 7 to 96% [15]. In the future, the gains in SNR provided by the ERC-PAC could enable protocol modifications to either improve spatial resolution or to shorten acquisition time, potentially allowing higher patient throughput.

We found overall improved image quality on T2WI and high b-value (b2000) DWI performed using a rigid ERC-PAC compared to PAC alone with mixed results for low b-value (b50) DWI and ADC. Regarding T2WI, our findings are similar to prior studies that reported improved image quality [9, 23] and visibility of anatomical details [7] for ERC-PAC. However, in the studies by Heijmink et al. [7] and Gawlitza et al. [23], the T2WI sequence was optimized for ERC-PAC and not the PAC, which could have contributed to these results [7]. However, in a study with closer matching of the T2WI parameters, there was no difference in overall image quality scores for 2 observers for PAC and ERC coils (*p* = 0.555) [8]. Regarding DWI, we found superior image quality for high b-value DWI using ERC-PAC for both observers, while Barth et al. found superior image quality only for one observer. In their study, only b1000 images were evaluated, whereas we evaluated b2000 images, which could explain the difference [8]. Ultimately, higher image quality for T2WI and high b-value DWI are more important than for low b-value DWI, as these sequences are key determinants of overall PI-RADS scoring.

The central question in our study was to determine if the use of the rigid ERC-PAC resulted in a meaningful difference in PCa detection rates, which we did not find for two observers with similar levels of experience interpreting prostate MRI. In an effort to reduce bias, we randomized the order of acquisition for sequences obtained without and with ERC and matched acquisition parameters exactly, which were key limitations in prior studies that found ERC-PAC superior to PAC [6, 8]. Comparison of our results with prior published studies on this topic again underscores that detection rates can be impacted by study design and acquisition parameters. Overall, our lesion detection rates for each observer for sequences obtained using the ERC-PAC and PAC are within the reported literature [24]. Other studies have found improved performance for ERC MRI [6, 7]. Superior sensitivity (0.76 vs. 0.45) and PPV (0.80 vs. 0.64) were found for acquisitions using a balloon inflatable ERC-PAC compared to PAC in a study of 20 men with prostatectomy correlation, however, there were key methodological differences in their study compared to ours such as differences in the in plane resolution and the number of channels used for the anterior coils employed for each acquisition [6]. In a separate study of 49 patients, the ERC-PAC protocol demonstrated superior sensitivity (78%) compared to non-ERC MRI for PCa detection, both with a “standard” (43%) (*p* < 0.0001) PAC protocol with matched acquisition parameters or an “augmented” (60%) (*p* < 0.01) PAC protocol using a greater number of signal averages [10]. Other studies have shown mixed or negative results [9, 11, 23]. For example, PCa detection rate was found to be superior using ERC-PAC only for one observer [PAC 69% (33/48) vs. ERC 86% (56/65) (*p* = < 0.05)] in one study [23].

The purported benefits of using a rigid ERC with a smaller diameter include the fact that rigid coils result in less prostate deformation, avoiding uncomfortable rectal distension, susceptibility artefacts due to the inflated balloon coil and the requirement for balloon inflation or perfluorocarbon [25]. We found that despite using a rigid ERC design, the majority of patients report discomfort at the time of coil insertion and during the exam with the coil in place, consistent with prior reports [7, 9]. Our findings agree with prior studies reporting that the greatest degree of discomfort occurred at the moment of coil insertion for both rigid and balloon inflatable ERCs [8, 26]. Of note, in a comparison study of different ERC devices, the rigid ERC was found to be more comfortable that the balloon inflatable ERC [26]. Our finding that despite the discomfort, the majority of patients still reported preferring the use of an ERC should improved PCa detection rates be achieved was also reported in the study by Baur et al. [9].

We recognize several limitations to our study. We have a small number of patients and lack of prostatectomy correlation in all but one patient. However, our recruited patients and results are reflective of our clinical practice. We also note that there was a wide range of time intervals between MRI and biopsy and that re-review of the pathology slides was not possible. Unfortunately, Gleason score can be under-estimated based on biopsy. We could not address the accuracy of cancer staging using ERC-PAC given lack of prostatectomy correlation. Another limitation is that we could not perform an intra-individual comparison DCE-MRI, since it was not feasible to repeat this sequence in the same setting. Blinding of the study acquisitions (ERC-PAC vs. PAC) for the qualitative imaging evaluation was not possible due to visibility of the ERC on the MRI images and could have potentially influenced the observers. Finally, there are disadvantages of using the ERC-PAC, including the time required for coil insertion, the need for an optimized clinical workflow to ensure appropriate device sterilization and storage, and cost considerations, which we did not assess.

## Conclusions

In our study, we exactly matched sequence acquisition parameters between ERC-PAC and PAC sequences and randomized the order of scanning, which allowed us to attribute differences in image quality, lesion detection and characterization to the presence of the rigid ERC, rather than differences in protocols, sequence parameters or patient fatigue. We found that 3 T MRI of the prostate performed using a combined rigid ERC-PAC demonstrates good diagnostic performance for detection of prostate lesions with reasonable agreement. While increases in eSNR and overall improved image quality were found using ERC-PAC when acquisition parameters are kept constant, the added benefit for PCa detection is not clearly demonstrated. Patient discomfort was also considerable, which must be taken into consideration when selecting acquisition methods, protocols and ERC devices for prostate imaging. Based on these results, we cannot currently support the routine use of the ERC-PAC in clinical practice. ERC-PAC could have a promising role for select problem solving cases and/or a tailored ERC protocol with an optimized workflow could enable reductions in overall scanning time with improved patient throughput, which warrant investigation in a larger number of patients.

## Supplementary Information

**Additional file 1.**

## Data Availability

All data generated or analysed during this study are included in this published article.

## Abbreviations

ADC: Apparent diffusion coefficient
CR: Contrast ratio
DCE-MRI: Dynamic contrast enhanced magnetic resonance imaging
DWI: Diffusion weighted imaging
ERC: Endorectal coil
ERC-PAC: Endorectal coil and phased array coil
IQ: Image quality
MpMRI: Multiparametric magnetic resonance imaging
MRI: Magnetic resonance imaging
PAC: Phased-array endorectal coil
PCa: Prostate cancer
PZ: Peripheral zone
PI-RADS: Prostate imaging reporting and data system
PSA: Prostate specific antigen
PSAD: Prostate specific antigen density
ROI: Region of interest
SD: Standard deviation
SI: Signal intensity
SNR: Signal-to-noise ratio
T2WI: T2-weighted imaging
TZ: Transition zone
