# Supplementary material for: Prostate MRI using a rigid two-channel phased-array endorectal coil: comparison with phased array coil acquisition at 3 T
Source: Cancer Imaging. 2022 Mar 16;22:15. doi: 10.1186/s40644-022-00453-7 (PMC8925156; doi:10.1186/s40644-022-00453-7)

**Supplemental Material**

*Coil Design*

The rigid ERC is a dual-channel receive-only phased array constructed with capacitively decoupled receive elements. Each 8.5 cm long receive element wraps around a cylindrical former, and the elements occupy the space along the entire length of the head of the probe. Detune circuits present on each of the elements prevent large currents during the transmit phase of the MRI system and capacitors on the elements are used for tuning the elements to the desired resonance frequency. A final capacitor matches each element to a preamplifier. The cable balun and preamplifiers are housed in the handle of the ERC. High SNR is due to the second channel, decouplers on the loop and the proximity of the preamplifier to the loops. The housing mimics an ultrasound probe with the maximum diameter at the head of the probe being 2.5 cm and reducing to 1.6 cm around the area where the patient’s anus would be present.

The disposable coil is also a two channel receive coil. The loops are geometrically decoupled from each other and wrap around a cylindrical former. Similar to the rigid ERC, the loops occupy the entire length of the head of the probe. The coil is connected to an interface box that has the necessary electronics to detune the coil in the transmit mode and amplify the signal in the receive mode. Since the loop is away from the preamplifier, there is signal loss and thus has a lower SNR when compared to the reusable Endorectal coil. This coil, however, is disposed of after one use.

**Supplemental Figure 1.** The image shows a reusable rigid two-channel receive ERC device.


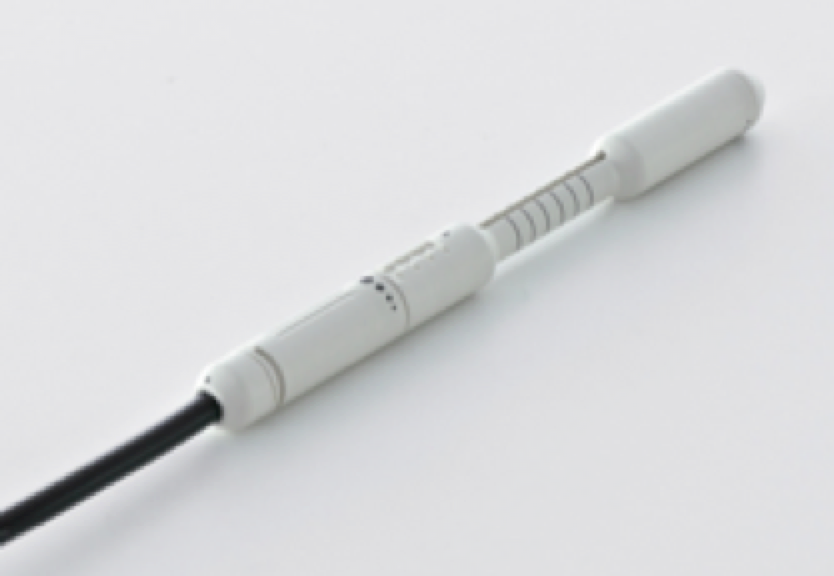

Supplement: Supplementary file 1 — Additional file 1. [file 40644_2022_453_MOESM1_ESM.docx]
